# Computer Simulations of Static and Dynamical Properties of Weak Polyelectrolyte Nanogels in Salty Solutions

**DOI:** 10.3390/gels4010002

**Published:** 2017-12-27

**Authors:** David Sean, Jonas Landsgesell, Christian Holm

**Affiliations:** Institute for computational physics, University of Stuttgart, Allmandring 3, 70569 Stuttgart, Germany; david.sean@icp.uni-stuttgart.de (D.S.); jlandsgesell@icp.uni-stuttgart.de (J.L.)

**Keywords:** computer simulations, electrophoresis, molecular dynamics, reaction ensemble Monte Carlo, weak polyelectrolytes, nanogels

## Abstract

We investigate the chemical equilibria of weak polyelectrolyte nanogels with reaction ensemble Monte Carlo simulations. With this method, the chemical identity of the nanogel monomers can change between neutral or charged following the acid-base equilibrium reaction HA ⇌ A^−^ + H^+^. We investigate the effect of changing the chemical equilibria by modifying the dissociation constant $K_{a}$. These simulations allow for the extraction of static properties like swelling equilibria and the way in which charge—both monomer and ionic—is distributed inside the nanogel. Our findings reveal that, depending on the value of $K_{a}$, added salt can either increase or decrease the gel size. Using the calculated mean-charge configurations of the nanogel from the reaction ensemble simulation as a quenched input to coupled lattice-Boltzmann molecular dynamics simulations, we investigate dynamical nanogel properties such as the electrophoretic mobility μ and the diffusion coefficient *D*.

## 1. Introduction

Micro- and nano-gels have recently gained increasing attention as intelligent materials [1,2,3,4,5,6]. Intelligent here refers to the fact that those materials are responsive to external stimuli. Therefore, intelligent employment of these materials can be used to achieve goals, like, e.g., drug delivery [7]. External stimuli can for example be temperature [8,9] or pH [1].

Polyacids typically become negatively charged in solution via the deprotonation of dissociable groups. The monomer charge is sensitive to the pH. In this work, a generic polyelectrolyte nanogel having a tunable acid dissociation constant is considered.

The electrophoretic mobility of colloidal particles with grafted weak polyelectrolytes have been theoretically treated on a mean-field level [10,11]. This work can help shed light onto the behavior of weak nanogels, which are purely polymeric. Simulations of nanogels have been performed mainly on strongly-charged gels [8,12,13,14,15,16,17,18].

Theoretical and computational work on weak polyelectrolytes has so far mainly focused on static equilibrium properties. Starting from the 1990s, computational methods were developed and employed to investigate the properties of weak polyelectrolytes. Using the constant pH method by Reed and Reed [19], the properties of weak polymers were studied starting from the 1990s [19,20,21,22,23,24]. Also in the 1990s, two different groups invented the reaction ensemble method [25,26]. This method allows one to simulate arbitrary reactions in chemical equilibrium [25,27]. The reaction ensemble is derived in a very straight forward manner from the grand canonical ensemble [27]. It has been employed previously to study properties of linear [28,29,30,31] and star-like [28,29] weak polyelectrolytes. Both methods are in general not equivalent [30] due to a different definition of the pH.

In a simulation, the definition of the pH is not obvious [30]. In the case of employing the constant pH method, the pH is a fixed input parameter without further meaning inside the simulation. This can yield the neglect of electrostatic screening. Additionally, the pH is treated as an implicit parameter independent of the volume. In contrast to this, the reaction ensemble method treats the pH as the concentration of H^+^ ions in the system. This yields the (experimentally observed) concentration dependence of the dissociation, which is in contrast to the constant pH method [30]. A more detailed comparison of the constant pH method, the reaction ensemble method in its usage modes by Landsgesell et al., can be found in [30].

For linear weak polyelectrolytes, the mean monomer charge along the backbone is generally not homogenous. The simple reason is that the monomers near the ends are, on average, at greater distances to the remaining monomers. As a consequence, they can increase their charge with a lower electrostatic energy penalty compared to monomers near the center of the chain. As such, weak polyelectrolytes generally are more charged at the ends. This effect has been predicted by theoretical arguments [32] and Monte Carlo simulations [21,28]. Interestingly, a similar end-effect can be observed for strongly-charged linear polyelectrolytes in a salt bath by considering effective charge [33]. Ion condensation-like effects regulate the charge of a strong polyelectrolyte in a manner similar to the way the charges self-regulate for a weak polyelectrolyte.

In general, the polymer topology can affect the dissociation equilibrium of the individual monomers quite severely. For example, the central node connecting the arms of a weak star-polymer will ‘resist’ charging due to its relative proximity to the remaining monomers [28], and in weakly-charged dendrimers, the outer charges are more dissociated than the inner charges [34,35].

In high salt conditions, the electrophoretic mobility is highly sensitive to the charge near the ends of a linear polyelectrolyte [36,37]. There is a negative relationship between the contribution of a given monomer charge to the mobility of the polymer and the overall proximity of this monomer and to the remaining ones [38,39]. For a linear polymer, this leads to a mobility end-effect akin to the charge regulation end-effect.

Although the charges of all monomers contribute to the total polymer mobility, their individual contributions are not equally weighted. Constraints imposed by the polymer configuration (e.g., proximity to crosslinks) affect the given weight of a monomer. For a weak polyelectrolyte, this picture is further complicated by that fact that the mean monomer charge itself is sensitive to a similar distance metric.

Since dynamical properties of weakly-charged nanogels have not been addressed theoretically, we will investigate in this work for the first time the electrophoretic mobility and the diffusion constant of the nanogel. The charged monomers of the ionized nanogel can be driven by the application of an external field *E*. The free solution electrophoretic mobility μ is defined as the polymer’s steady-state velocity *v* scaled with the magnitude of the driving field μ=v/E. The nanogel’s steady-state velocity is then determined from the force balance between viscous drag and the externally-applied force.

Electrostatic attraction causes counter-ions in solution to preferentially crowd near the charged monomers. The mean field description of the counter-ion concentration around a charged monomer has an exponential decrease over the so-called Debye length $\lambda_{D}$ that characterizes the extent of the counter-ion cloud and the range over which electrostatic interactions are effective.

Since the counter-ions have an opposite charge as the backbone monomers of the gel, the electric field *E* creates on both ion species forces in opposite directions. These forces cause a hydrodynamic shear over the length scale of $\lambda_{D}$. This shear effectively cancels the long-range hydrodynamic perturbation between the charged monomers. Thus, the electrically-driven motion of the charged monomers of the nanogel are screened from the other monomers, resulting in what appears to be a free-draining behavior.

This effect is modulated by the distribution of the ionic cloud near the monomers. This distribution is affected by the local charge density of the monomers, which is a direct expression of the mean dissociation state. Thus, the amount hydrodynamic screening effect between the monomers can vary inside the nanogel. We therefore need to turn to explicit simulations in order to investigate the electrophoretic mobility.

Although the main dynamical property we investigate is the electrophoretic mobility, we also investigate how diffusion is affected by salt concentration and dissociation constant. We utilize the components of the nanogel trajectories orthogonal to the applied field to obtain the nanogel diffusion coefficients.

**Figure 1:**
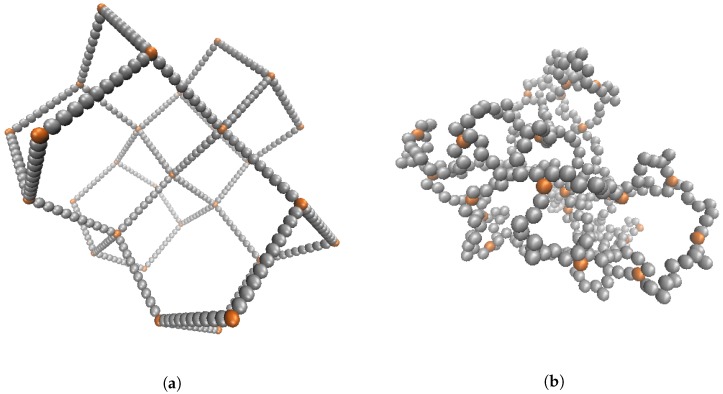
(Color online) Simulation screenshot of the initialized state of 29 crosslinks attached with 40 polymers having 10 monomers in each chain for a total of $N_{0}=429$ simulation beads. Screenshots shown immediately after the initial placement of the beads (**a**) and after equilibration (**b**).

Our nanogel consists of a total of $N_{0}=429$ explicit particles, which are bonded to form a polymeric mesh similar to [12]. The nanogel is initialized with the crosslinking nodes placed on a diamond lattice. All nearest nodes are connected with linear polymer strands consisting of 10 monomers. The structure is given an overall spherical shape by deleting all beads beyond a specified cutoff distance to the nanogel center of mass. Once this preliminary structure is initialized, all dangling ends (nodes having zero or one connection) are deleted. This results in a gel with tetra-functional crosslinks in the interior. Nodes on the the periphery (near the outside) are connected to two or three polymer chains, as shown in Figure 1. Note that in this figure, the crosslinks are colored to highlight the topology, and the mobile ions are not shown. Although the crosslinks can have more bonded neighbors, they are identical to the nanogel monomers in all other respects. In this exploratory study, we consider an idealized model nanogel in order to facilitate the analysis and interpretation of an already rich system. We thus do not alter the topology of the nanogel, nor do we include polydispersity in the polymers connecting the nodes. More realistic nanogel models can be constructed following the methodology in [40,41].

We employ two distinct simulation approaches to evaluate the properties of the nanogel. The annealed charge distribution of the weak polyelectrolyte nanogel is investigated with the reaction ensemble method. This method generates an ensemble of nanogel states that have different conformations (monomer positions), as well as different chemical configurations (protonated/deprotonated monomer states). For each parameter combination considered, we generate an ensemble having a minimum of 3000 states. These are then successively used to obtain static quantities like the mean nanogel size or the mean nanogel charge.

However, the reaction ensemble method is ill-suited to investigate dynamical quantities since it employs Monte Carlo moves, which do not follow a physical kinetic path. Therefore, the dynamical quantities are investigated with separate runs of pure MD simulations that perform an explicit time integration. Conversely, these NVT simulations are not designed to included changes in the charge state of our chemical species.

As a consequence of these limitations, we thus confine our dynamic investigations to “quenched” weak polyelectrolytes. Since a change in the chemical species of the monomers only affects the charge (all other properties remain unaffected), we can perform a charge pre-average of the *i*-th monomer $q_{i}=\langle q_{i}\rangle$. During the pure MD simulations, the monomer charges have a static value corresponding to the ensemble average sampled from the annealed reaction ensemble results.

Other simulation studies have combined the reaction ensemble method with MD as a means to sample dynamical properties [42,43,44]. The hybrid dissipative particle dynamics (DPD) simulations of [42] have separated the simulation volume to include reaction chambers where the chemical reactions are allowed to take place. The reactants and products are allowed to diffuse in and out between the reaction chamber and zone where chemical reactions are not allowed. Since this latter zone can be in chemical equilibrium without explicit reactions, it is used in order to probe dynamical properties. The polymer growth simulations of [43,44] use the reaction ensemble method with DPD to simulate the polymerization process. They employ the fractional particle concept to vary the number of particles in a continuous manner such that the dynamical trajectories remain deterministic.

The paper is organized in the following way. In the next section, we present the static results obtained from the reaction ensemble Monte-Carlo method, followed by a dynamical results obtained from molecular dynamics simulations. We discuss the results in the their respective sections.

In the section that follows, we describe the methods we apply, namely the reaction ensemble Montel-Carlo and the molecular dynamics method. Lastly, we present a summary of the main results and our concluding remarks.

## 2. Results and Discussion

### 2.1. Static Properties

The reaction ensemble Monte Carlo simulations provide all the static properties shown in this section.

#### 2.1.1. Monomer Charge

**Figure 2:**
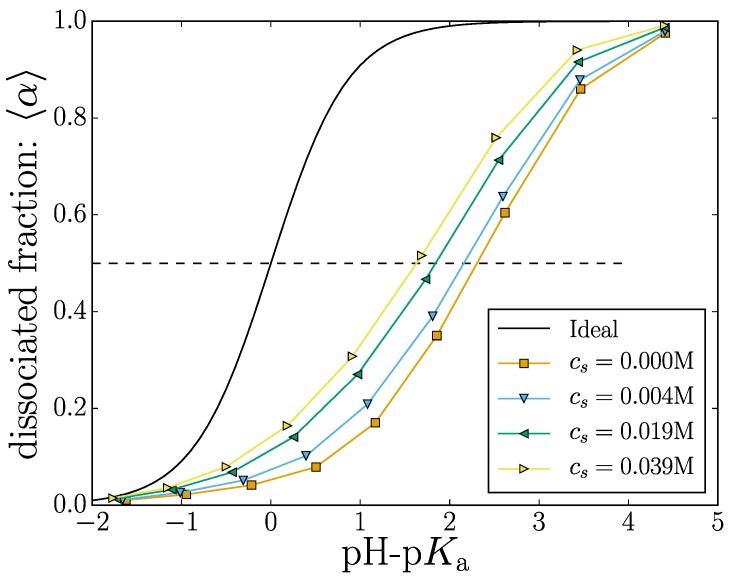
(Color online) Titration curve: The mean fraction of dissociated monomers versus pH-p*K*_a_. The different markers indicate different salt concentrations. The ideal titration curves is shown as a solid black line.

The titration curve shown in Figure 2 shows the effect of changing the dissociation constant $K_{a}$ on the mean degree of dissociation.

We define the latter as:

(1) $$\begin{array}{c} \langle \alpha \rangle =\frac{\langle \Sigma_{i=1}^{N_{0}}\alpha_{i}\rangle }{N_{0}}=\frac{\langle N_{A}\rangle }{N_{0}}, \end{array}$$

where the $\alpha_{i}$ is the state of the *i*-th monomer, 〈·〉 denotes an ensemble average, $N_{0}$ is the number of monomers in the nanogel and $N_{A}$ is the number of deprotonated $\begin{array}{c} A^{-} \end{array}$ monomers. Since a dissociated monomer carries a (negative) unit charge ($\alpha_{i}=\left|q_{i}\right|$), this metric can also be used to describe the mean nanogel charge. Increasing the value of *K*_a_ will yield an almost completely charged nanogel.

Adding salt ions into the solution screens the electrostatic interactions, effectively reducing the electrostatic potential energy between two charged monomers. This screening results in an increased number samples where the monomers are dissociated for a given value of *K*_a_. Thus, the curves in Figure 2 are shifted towards the ideal curve result (where monomers do not interact) with increasing salt content. Our results demonstrate the general tendency that increasing the electrostatic screening will result in more charged groups along the polymer backbone. This is well known and has been theoretically described [45].

**Figure 3:**
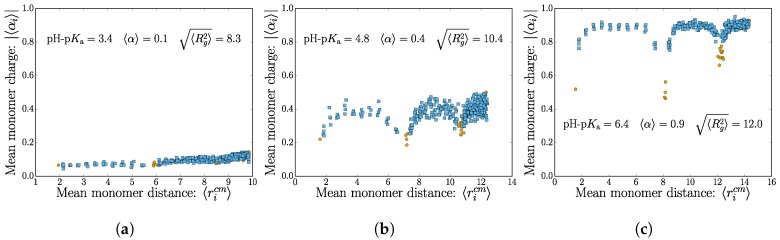
(Color online) The mean monomer dissociation state $\langle \Sigma_{i}\alpha_{i}\rangle$ as a function of the mean distance to the nanogel center of mass. The different subplots (**a**–**c**) show an increasing value of the dissociation constant $K_{a}$. The monomers belonging to a crosslink are colored in orange. All cases shown here have the same salt concentration of $c_{s}=0.004$ M.

There are two important length scales in play: the nanogel size $\sqrt{\langle R_{g}^{2}\rangle }$ (explored in detail in the next section) and the mean polymer extension, which probes the nanogel structure. Using the intermediate salt concentration $c_{s}=0.004$ M, we are now taking a finer look and consider the individual monomer contributions to the total nanogel charge by considering the mean monomer dissociation state $\langle \alpha_{i}\rangle$. This is shown for three different values of pH-p*K*_a_ in Figure 3, where we choose to use the mean monomer distance from the nanogel center of mass as the abscissa.

The plot shows how the charges are distributed for weak polyelectrolyte of increasing mean dissociation state 〈α〉. Note that there is an expected increased density of points for distal monomers (as is expected for a homogenous monomer distribution in a spherical nanogel). Crosslink monomers are colored differently to highlight the topological effects of charge repulsion that occurs near the nodes.

In the low mean dissociation state, the nanogel topology does not stand out. The monomers are only weakly charged on average, and thermal fluctuations cause internal conformational changes, which “wash-out” the internal polymer structure. There is a slight increase in dissociation for monomers found far from the center of mass. This is to be expected since the electrostatic potential is smallest near the nanogel surface.

The swelling caused by the higher monomer charges in the moderate dissociation state allows for the internal connectivity to be manifested. Figure 3b shows that monomers near the crosslinks have a lower tendency to dissociate. The lowest dissociation state typically belongs to the crosslinks themselves. The tendency for the charges to be found at the nanogel surface remains apparent here as an envelope over the profile from the nodes.

Lastly, the nearly fully-dissociated polyelectrolyte case contains striking features of the internal structure. The polymer chains between the crosslinks are essentially fully charged, except for the crosslinker monomer itself and one neighboring monomer. Again, there is a noticeable increase in monomer charge near the surface of the nanogel.

#### 2.1.2. Nanogel Size

**Figure 4:**
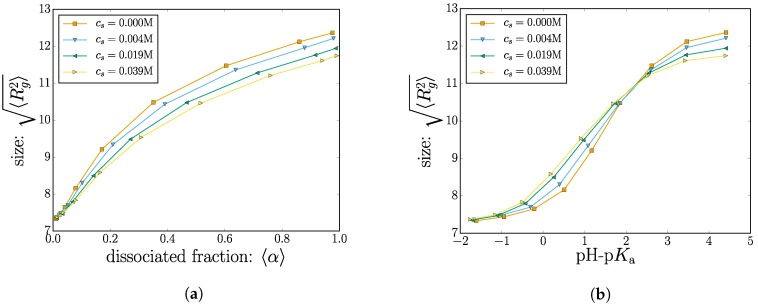
(Color online) (**a**) The radius of gyration is plotted as a function of the mean degree of dissociation; (**b**) the radius of gyration is plotted as a function of pH-p*K*_a_. The different markers indicate different salt concentrations.

As discussed above, the dissociated monomers carry a negative unit charge. An increase in the electrostatic repulsion between charged monomers causes an increased radius of gyration. This is shown in Figure 4, where we plot the root-mean-squared radius of gyration $\sqrt{\langle R_{g}^{2}\rangle }$.

Our results of Figure 2 indicate that the electrostatic screening increases the the net dissociation (and therefore nanogel charge). A high net charge is expected to provoke electrostatic swelling of the nanogel. However, the screening effect from the salt would also reduce the swelling caused by electrostatic repulsion between charged monomers. Since these two influence the polymer size in different directions, the question of how the screening changes the polymer size (if at all) is not obvious. The two limiting cases are discussed separately below.

One can consider the extreme of infinitely small Debye length with “perfect” screening. This will eliminate the effect of the electrostatic swelling and increase the amount of dissociated monomers. Thus, the majority of monomers will become charged (depending on the dissociation constant) without an associated increase in the polymer size. In this situation, the polymer charge increases with $K_{a}$ with negligible effects on the nanogel size.

On the other extreme with zero screening (infinite dilution of the monomer’s counter ions), having two charged monomers on the chain comes at a high energetic cost. Increasing the value of $K_{a}$ will eventually overcome this cost, and the most distal units (polymer ends) will become charged, which will result in a rod-like conformation. The electrostatic repulsion from these ends will yield a conformation akin to a polymer being pulled by the two ends. Further increases in $K_{a}$ will give rise to an accentuated tendency towards rod-like conformations. In this scenario, the polymer size increases with the amount of charged units until it reaches the rod-like saturation size.

To explore this scenario, results with different salt concentrations are also plotted in Figure 4a. As expected, when the charge fraction is low (〈α〉≈0.1), the effect of adding salt is marginal since there are very few charged monomers with an electrostatic repulsion to screen.

Consider now a moderately-charged nanogel (〈α〉≈0.4 in Figure 4a). A group of four data points (corresponding to different salt concentrations) with $\sqrt{\langle R_{g}^{2}\rangle }\approx 10.5\sigma$ all correspond to nanogels having the same dissociation constant $K_{a}$. The reduced electrostatic energy increases the likelihood of sampling monomers in the charged dissociated state: increasing the salt concentration increases 〈α〉≈0.4. Increasing mobile salt ions also affects the conformations by reducing the electrostatic repulsion between monomers. The two effects cancel out, and for all salt concentrations explored, the nanogels have the same size despite having different charges.

The other extreme, for situations where almost all of the monomers are charged (〈α〉≈1 in Figure 4a), adding salt ions no longer increases the polymer charge since this mechanism has saturated. In this region, the effect of adding salt decreases the radius of gyration by reducing the electrostatic swelling.

As an alternative depiction, the nanogel size is plotted as a function of pH-p*K*_a_, shown in Figure 4b. Two distinct regimes become apparent as a crossover at a critical value of pH-p*K*_a_. On the left side of the crossover, increasing the salt concentration will increase the polymer size, whereas the opposite is true for higher values of pH-p*K*_a_ beyond the crossover.

We were not able to reproduce this effect for a few cases of linear polymers and star-polymers. It appears that polymer connectivity via the crosslinks is an important factor in creating this crossover effect. The non-monotonic behavior of the size as a function of salt concentration is consistent with previously-published data [46,47].

#### 2.1.3. Ion Distribution

Counterions (including salt cations) from solution are attracted to the negatively-charged nanogel. Counterion condensation along the polymer backbone is typically considered to take place when the polymer linear charge density meets the Manning criterion. For an infinitely long rod-like polyelectrolyte having a uniform linear charge density, the criterion is met when the linear charge density is such that the mean distance between unit charges along the backbone is less than the Bjerrum length, $\ell_{q}<\ell_{B}$, where $\ell_{q}$ is the distance between unit charges.

We demonstrated in Figure 3 that the monomer charge is not uniform in the nanogel; it depends on both the topological features of the internal polymer structure, as well as the distance to the nanogel center. Nonetheless, we find it instructive to consider an effective linear charge density by assuming the nanogel is composed of a uniformly-charged linear polyelectrolyte having a total charge $eN_{0}\langle \alpha \rangle$. This coarse metric for the effective linear charge density can be used as a guide to help draw a picture of how the ions are distributed in and around the nanogel. In our model, the distance between monomers is σ; thus, the effective distance between uniform charges is the ratio between the total “linear polymer contour length” and the total nanogel charge:

(2) $$\begin{array}{c} \ell_{q}=\frac{N_{0}\sigma }{N_{0}\langle \alpha \rangle }. \end{array}$$

The dimensionless Manning parameter thus takes the form of $\langle \alpha \rangle \ell_{B}/\sigma$ and when evaluated with our value of $\ell_{B}=2\sigma$ gives favorable conditions for counterion condensation when 〈α〉>1/2. A dotted line is conveniently included in Figure 2 as an estimation of where this threshold is crossed for the simulated nanogel.

**Figure 5:**
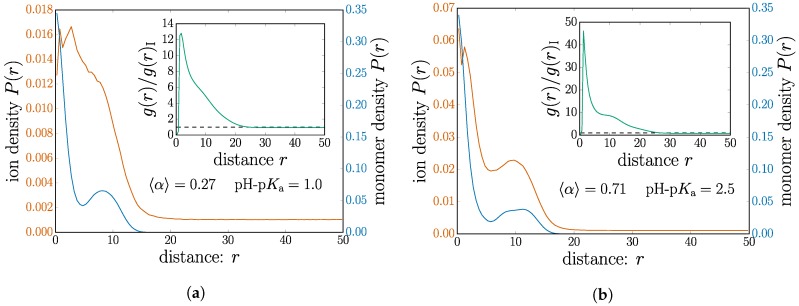
(Color online) The distributions of ions and monomers around the nanogel center of mass. The inset shows the monomer-ion pair correlation function g(r) between the monomers and the mobile ions. The two cases are chosen (**a**) above and (**b**) below the Manning parameter. Both subplots have the same salt concentration $c_{s}=0.019$ M.

We select two nanogel cases on opposite sides of the threshold and plot the distribution of monomers and mobile ions in Figure 5. For ease of comparison, the two cases have the same salt concentration of $c_{s}=0.019$ M. The nanogel chosen below the threshold, shown in Figure 5a, has a mean dissociation of 〈α〉=0.27. The monomer profile measured from the nanogel center of mass is plotted together with that of the mobile ions. We choose to use separate *y* axes for these data to optimize the visual representation of their features since the scales are quite different.

In both cases shown in Figure 5a,b, the monomer profile shows a decrease in density followed by a small peak near the nanogel surface, a feature of the internal structure. Although both plots have the same scale for the monomer density, inspection of the nanogel size by the monomer coverage on the *x*-axis shows an obvious swelling for the higher charged case; see the corresponding data points in Figure 4.

Although the monomer profile shows a simple electrostatic swelling, the ions near the nanogel exhibit quite different behavior. Indeed, crossing the manning threshold radically changes the way in which the ions are distributed.

In the case of Figure 5a, below the Manning criterion, the ions are without a doubt attracted to the nanogel neighborhood. There is a higher concentration of ions inside the nanogel than in the bulk. Although the peak seems to coincide with the dip in monomer density, ionic concentration shares features of the nanogel for length scales comparable to the nanogel size. They behave as a cloud of mobile ions hovering in the vicinity of the nanogel center.

In contrast to this, the ionic density shown in Figure 5b for 〈α〉=0.71 shows that the ions share features remarkably similar to the monomers. This situation, chosen above the Manning criterion, demonstrates a structure for the ions, which correlates closely with the monomer structure. This is consistent with the picture of ions condensed on the polymer backbone.

Since there is no obvious method to count the number of condensed counter ions, some authors can choose to simply tabulate the counter ions inside some arbitrary cutoff distance to the monomers [33,48]. In order to further verify this, we look at the pair correlation function g(r) only measured between monomer-ion pairs. These data are shown as insets in Figure 5 for their corresponding plots. Counterion counting simply amounts to integrating g(r) up to a chosen cutoff distance. Over short distances, inspection of the absolute value of g(r) between the two cases demonstrates a significantly higher number of ions for the case where Manning condensation is expected. This demonstrates increased counterions without imposing a cutoff.

Furthermore, the shape of the g(r) insets also relays information about how the ion distribution differing between the two cases. There is an apparent “shoulder”, which is much more pronounced in Figure 5a,b. The classical Poisson–Boltzmann description of a charged rod predicts an exponential decrease. In a nanogel, we expect some kind of shoulder on the exponential decrease arising from different branches of the polymer network. This distortion to the Poisson–Boltzmann picture should roughly span the nanogel size. The correlations between monomers are expected from by the imposed connectivity; the sharper shoulder in the inset of Figure 5b suggests that an ion correlation mediated the monomers. This is precisely shown in the main plot of Figure 5b.

**Figure 6:**
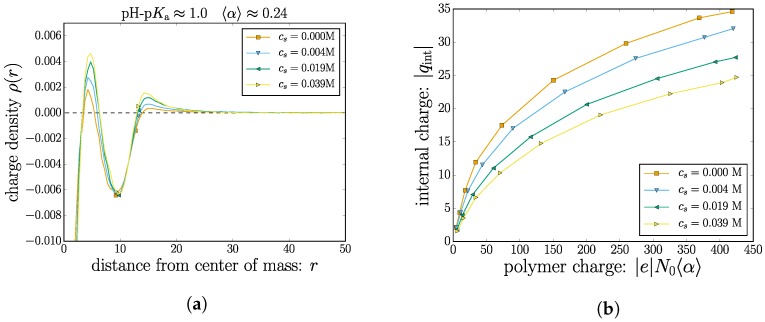
(Color online) (**a**) For the selected case corresponding to 〈α〉≈0.24, the total charge density is plotted as a function of the radial distance to the nanogel center of mass. The symbols mark (in ascending order) the value of $R_{H}$ and critical radial position $r^{*}$ at which 100% of the nanogel monomers are included. (**b**) The integrated net charge of the nanogel complex (up to $r^{*}$) as a function of pH-p*K*_a_.

We now turn to combining the monomeric and ionic contributions to the nanogel charge. Figure 6a shows an example of the total charge density around the nanogel center of mass for a case with 〈α〉≈0.24. The plot includes two sets of markers: one at $r=R_{H}\approx 10$; and one at a critical distance $r^{*}\approx 13$. If one is interested in the total charge in the volume spanned by the nanogel, the charge density ρ(r) needs to be integrated up to a specified radial distance. Clearly, using $R_{H}$ (or the numerically similar $R_{g}$) is insufficient. The plot demonstrates that important features (oscillations) of the charge density will not be integrated into the total charge of the nanogel complex. This behavior is consistent throughout all cases studied, although not all of them have $R_{H}$ coinciding with a local minima. To remedy this, we first find the critical radial distance $r^{*}$ defined as the minimum value such that integrating the monomer density in the range $r\in (0,r^{*}$ yields the total number of monomer beads $N_{0}$. In other words, the complete nanogel is critically captured in a sphere of radius $r^{*}$.

Using this value of $r^{*}$ as an integration limit allows one to define the internal nanogel charge:

(3) $$\begin{array}{c} q_{\mathrm{int}}=\int_{0}^{r^{*}}\rho \left(r\right)dr. \end{array}$$

In Figure 6b, the total internal charge $q_{\mathrm{int}}$ is plotted as a function of the total monomeric charge.

For the case with no added salt $c_{s}=0$ M, the mobile counterions regulate a portion of the monomer charge, but since a fraction of the mobile counterions leak out into bulk solution, there remains a net nanogel charge. The propensity to compensate for the monomeric charges increases with increasing salt concentration.

### 2.2. Dynamic Properties

We now turn to dynamical properties of the nanogel, mainly its diffusion coefficient *D* and its electrophoretic mobility μ. These transport coefficients are obtained from pure MD simulation with hydrodynamic interactions. The static monomer charges are taken as their mean values from the RxMC simulation results. Charged polyelectrolytes undergoing electrophoretic migration are said to be free-draining, i.e., the friction coefficient ζ follows the Rouse prediction $\zeta ∼N_{0}$, which comes at a stark contrast with the Zimm result, which scales with the polymer size.

Due to the electrohydrodynamics at play, the Nernst–Einstein relation $D=\mu k_{B}T$ is expected to fail for strong polyelectrolytes undergoing electrophoretic migration [48,49]. This entails that the diffusion coefficient cannot be obtained by simple measurements of the mobility or vice versa.

However, these physical effects are uncoupled: the diffusion in the direction orthogonal to the driving field follows the Zimm prediction. We can thus extract these independent metrics from the same simulation trajectory; provided the driving field does not significantly deform the nanogel [50,51].

#### 2.2.1. Diffusion

**Figure 7:**
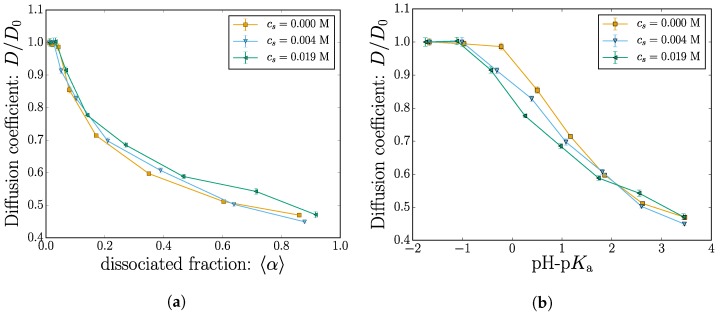
(Color online) The diffusion coefficient *D* plotted as a function of (**a**) the dissociated fraction and (**b**) pH-p*K*_a_. In all cases, the *y*-axis is rescaled with the value at lowest dissociation α ≈ 0 or pH-p*K*_a_ ≈ −2.

The nanogel diffusion is measured using the mean-squared-displacement (MSD) in the *y* and *z* direction. The MSD is fitted with a relation of the form:

(4) $$\begin{array}{c} \Delta y^{2}+\Delta z^{2}=4D\Delta t \end{array}$$

where the diffusion coefficient *D* is used as a fitting parameter. The fitted results are shown in Figure 7a as a function of the mean dissociation fraction and in Figure 7b as a function of pH-p*K*_a_. In both subplots, the *y*-axis is rescaled with the diffusion value measured from the first point, i.e., α≈0 and pH-p*K*_a_ ≈ −2.

The diffusion coefficient decreases with the nanogel charge. This qualitative behavior is consistent with the observed swelling shown in Figure 4. Within the uncertainty of the fitted data, the simulations at different salt concentrations only marginally affect the diffusion coefficients. The expected qualitative decrease of the diffusion as the nanogel size increases can be observed. Close inspection of the Figure 4b where the diffusion is plotted as a function of pH-p*K*_a_ reveals signs of the size crossover observed in Figure 4 near pH-p*K*_a_ ≈ 2. The diffusion data fitted from the MSD are unfortunately more noisy, and the apparent crossover is not as convincing as from the static data.

#### 2.2.2. Electrophoretic Mobility

**Figure 8:**
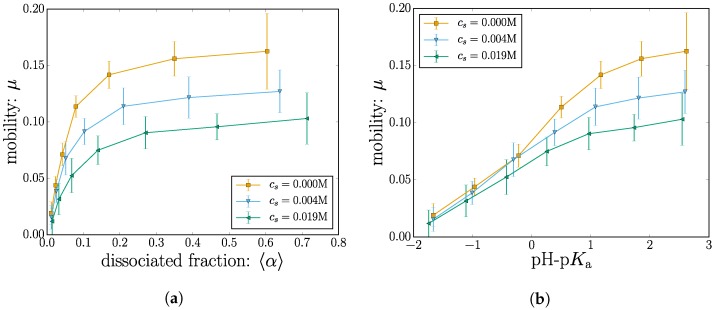
(Color online) The electrophoretic mobility is plotted as a function of (**a**) the dissociated fraction and (**b**) pH-p*K*_a_.

The nanogel velocity is measured by considering the total displacement in the *x* direction for the duration Δt of the simulation v=Δx/Δt. The mobility is thus given by rescaling the velocity with the value of the driving field μ=v/E. The measured mobility is plotted in Figure 8 both as a function of the dissociated fraction 〈α〉 and pH-p*K*_a_.

At low charge, the mean nanogel position does not significantly drift in the direction of the applied field. As the nanogel becomes charged by increasing pH-p*K*_a_, the mobility rises accordingly and ultimately shows signs of leveling off.

At high values of pH-p*K*_a_, some degree of leveling off is to be expected since the effect of changing the dissociation constant should saturate to the state where all the monomers are charged. However, recall that the parameter space has been reduced due to the computational cost of these simulations; the simulation data terminate before the saturation point is reached.

When plotted as a function of 〈α〉, the mobility shows a surprisingly early plateau. This is especially true for the case with the higher salt concentration $c_{s}=0.019$ M. Inspection of this situation reveals that the mobility at a monomer charge fraction of 〈α〉≈0.28 is remarkably similar to the mobility at a monomer charge fraction of 〈α〉≈0.7; despite having a significantly higher charge.

The conventional interpretation of a constant velocity is to consider a force balance between the effective charge and the effective friction. Although these loose notions of effective need to be defined for a specific context, the idea here would be that since the charge grows, some kind of effective friction grows in the same manner to balance out the forces.

Ions that enter the nanogel can partially compensate for the nanogel charge, as shown in Figure 6. In addition to that, they also take part in local hydrodynamic shearing, which affects the nanogel friction. Since increasing the nanogel charge also contributes to a growing number of H^+^ ions, these effects can come into play in a nontrivial manner. We are currently exploring the physical mechanisms behind the observed plateau in the mobility.

## 3. Materials and Methods

### 3.1. Reaction Ensemble Monte Carlo

Reaction ensemble Monte Carlo (RxMC) simulations provide a means to numerically consider chemical equilibrium [25,27]. The monomers of the nanogel can be dissociated according to the a chemical reaction:

HA ⇌ A^−^ + H^+^,

where a neutral monomer HA can dissociate into a negatively-charged species A^−^ and a free mobile ion H^+^. In general, the reaction follows:

(5) $$\sum_{i=1}^{z}\nu_{i}s_{i}=0$$

for *z* chemical species of type $s_{i}$ with stoichiometric coefficients $\nu_{i}$ [52]. The addition and removal of particles in the simulation volume follows the law of particle conservation:

(6) $$N_{i}=N_{i}^{0}+\nu_{i}\xi$$

where $N_{i}$ is the number of particles after a reaction, $N_{i}^{0}$ is the number of particles prior to a reaction and ξ is the reaction coordinate that characterizes the “extent” of the reaction. For a trial reaction, ξ is first randomly chosen between ξ=+1 for a deprotonation (forward) reaction and ξ=−1 for a protonation (backward) reaction. The transition probability for a forward reaction from state *k* to *l* in terms of an individual reaction step ξ=+1 with regard to detailed balance conditions is given by [27]:

(7) $$p_{k\to l}^{\xi }=\min\left\{1,{\left(\beta P^{0}V\right)}^{\bar{\nu }\xi }K^{\xi }\prod_{i=1}^{z}\left[\frac{N_{i}^{0}!}{(N_{i}^{0}+\xi \nu_{i})!}\right]\exp\left(-\beta \Delta U_{\mathrm{pot},\;k\to \;l}\right)\right\},$$

where *K* is the dimensionless dissociation constant; $P^{0}$ is the standard pressure; $\Delta U_{\mathrm{pot},\;k\to l}=U_{\mathrm{pot},\;k}-U_{\mathrm{pot},\;l}$ is the potential energy difference; *V* is the simulation box volume; $\bar{\nu }=\sum_{i}\nu_{i}$ is the change in the number of simulation particles; and $\beta =1/k_{B}T$ is the inverse thermal energy. The dimensionless dissociation constant is an input parameter, which can be calculated via:

(8) $$K=\exp\left(\frac{\sum_{i=1}^{z}\nu_{i}\mu_{i}^{0}}{k_{B}T}\right),$$

where $\mu^{0}$ is the chemical standard potential for each species. This dimensionless *K* is related to the dissociation constant $K_{a}$, which is known from the law of mass action (and which is typically not dimensionless) via:

(9) $$K_{a}=\prod_{i}c{\left(s_{i}\right)}^{\nu_{i}}=K{\left(\beta P^{0}\right)}^{\bar{\nu }},$$

where $c\left(s_{i}\right)=N_{s_{i}}/V$ denotes the concentration of species $s_{i}$. $U_{\mathrm{pot}}$ is the potential energy of the system [27]. Therefore, it is evaluated via the system particle positions according to the sum of the MD potentials described in the next section. In all simulations that employ the reaction ensemble, we do not apply an electric field, which would influence the potential energy, e.g., that the released protons have. Between the Monte Carlo reactions attempts, the system is permitted to evolve by performing a fixed number of MD simulation steps in the NVT ensemble. The goal of these MD steps is solely to sample the conformational partition function [53]. Therefore, Monte Carlo (MC) sampling could also be used instead of MD sampling of the configuration space. However, MD sampling has the advantage of not having to implement MC moves, which efficiently sample the configuration space of polymer gels [54].

### 3.2. MD Simulations

Molecular dynamics is used in two distinct areas in this work; (i) inside the RxMC as a means of exploring conformational phase space and (ii) as pure MD of the quenched nanogel.

Excluded-volume interactions are applied between all particles in the system: monomers; cations; anions and counterions. They are imposed via a truncated Lennard–Jones interaction of the form:

(10) $$\begin{array}{c} U_{\mathrm{WCA}}\left(r\right)=\left\{\begin{array}{cc} 4\epsilon \left[{\left(\frac{\sigma }{r}\right)}^{12}-{\left(\frac{\sigma }{r}\right)}^{6}\right]+\epsilon & \mathrm{for}\;r<r_{c}\\ 0 & \mathrm{for}\;r\geq r_{c} \end{array}\right. \end{array}$$

where ϵ is the well depth, σ is the bead diameter (the same for all particles), *r* is the distance between two particles and the cutoff distance $r_{c}=2^{1/6}\sigma$ is chosen such that the potential is purely repulsive [55]. The nominal particle diameter σ serves as the MD unit of distance, and the well depth ϵ is used as the unit of energy.

Monomers are bonded with finitely extensible nonlinear elastic (FENE) springs that have a potential of the form:

(11) $$\begin{array}{c} U_{\mathrm{FENE}}\left(r\right)=-\frac{1}{2}\;k_{\mathrm{FENE}}\;{r_{0}}^{2}\;\ln\left[1-\frac{r^{2}}{{r_{0}}^{2}}\right] \end{array}$$

where the spring constant $k_{\mathrm{FENE}}=30\epsilon /\sigma^{2}$ and maximum extension $r_{0}=1.5\sigma$ is chosen to prevent bond-crossing [56].

Coulombic interactions between particles *i* and *j* are implemented via a potential of the form:

(12) $$\begin{array}{c} U_{P3M}\left(r\right)=\ell_{B}k_{B}T\frac{q_{i}q_{j}}{r} \end{array}$$

where the Bjerrum length $\ell_{B}=2\sigma$ scales the interaction strength and (unless specified) each particle contains the same scaled charge |q|; essentially the valency. The Bjerrum length indicates the length where the electrostatic energy is or the order of the thermal energy:

(13) $$\begin{array}{c} \ell_{B}=\frac{e^{2}}{4\pi \epsilon_{0}\epsilon k_{B}T}. \end{array}$$

For typical conditions in water, this is about $\ell_{B}^{\mathrm{EXP}}\approx 0.71\;\mathrm{nm}$.

When used in the RxMC simulations, the particle positions are integrated following Langevin dynamics [55]. These computationally less expensive simulations neglect the hydrodynamic interactions, which do not affect static quantities. The effect of salt concentration is explored by adding a total of 0, 200, 1000 and 2000 salt ions pairs in the simulation box (corresponding to molar salt concentrations of $c_{s}=0$ M, $c_{s}=0.004$ M, $c_{s}=0.019$ M and $c_{s}=0.029$ M, respectively).

The typical titration results plot the resulting dissociated fraction α as a function of pH-p*K*_a_ (or some equivalent metric). In this work, we employ the naive definition of pH as $-\log_{10}\left(c\left(\begin{array}{c} H^{+} \end{array}\right)/c_{0}\right)$ where $c(\begin{array}{c} H^{+} \end{array})$ is the concentration (in molar units) of H^+^ ions released in the simulation volume by the nanogel, as well as p*K*_a_ = $-\log_{10}\left(K_{a}/c_{0}\right)$, where $c_{0}$ is a reference concentration. The chemical equilibrium constant $K_{a}$ is varied in order to achieve an approximate range of pH-p*K*_a_ between −2 and 5 that covers the ionization limits of the nanogel. In the simulations in this paper, this is achieved via sweeping the dissociation constant *K* (which equals a sweep of $K_{a}$ for fixed β and $P^{0}$). As a warning, we emphasize the following: sweeping the reaction constant is not equivalent to sweeping the pH of the solution [30].

Sweeping the dissociation constant is equivalent to exchanging the chemical nature of the reacting beads and assigning a different dissociation constant to them. The measured pH then is the eigen pH [30], which arises in the simulation box. Therefore, sweeping the dissociation constant is not equivalent to sweeping the pH where additional screening effects occur [30].

When performing the pure MD simulations of the quenched nanogel undergoing electrophoresis, hydrodynamic interactions are computed using the Lattice–Boltzmann method. Since these simulations are computationally more demanding, the range of explored pH-p*K*_a_ is narrow, and the high salt case of $c_{s}=0.029$ M is not explored.

The degree of dissociation of a gel depends on the electrostatic potential at a given position [45]. Under the influence of an external potential gradient imposed by electrodes at opposite ends of the bulk solution, the nanogel moves in the direction of lower potential energy. By virtue of this movement, the local external electric potential changes for the nanogel.

In our migration simulations, we impose a fixed degree of dissociation; as obtained from the RxMC simulation at a reference external potential. Our simulation models a situation where the differences in external potential do not significantly change the degree of dissociation.

This can be physically interpreted as a weak field or small displacement limit. Alternatively, migration methods like traveling wave electrophoresis [57] can achieve a constant electric gradient while maintaining a constant external potential local to the nanogel.

The effect of an externally-applied electric field is modeled on all beads as a constant force in the *x* direction proportional to the bead charge $q_{i}$. Thus, in addition to the effect of all potentials, the bead will migrate under the influence of the force $F_{x}=q_{i}E$, where the use of E=0.10 results in a field that does not significantly deform the polymer conformations.

Recall that the pure MD simulations of the quenched nanogel have monomer charges that are set to the average value taken from the RxMC simulations. This results in a floating-point value for the charges (or valencies) in Equation (12). In order to enforce electrostatic neutrality, we renormalize the monomer charges such that the nanogel contains an integer charge and add the corresponding number of counterions of unit valency. We do this to reduce complications arising from valency effects of the mobile charge carriers. Simulations are all carried our using the ESPResSo package in its developer version [58,59,60], using the P^3^M algorithm for electrostatic interactions [61,62] and the GPU lattice Boltzmann implementation [63] with correct thermalization spectrum [64].

All nanogels are simulated in a 100 × 100 × 100 cubic simulation box to minimize finite-size effects. Depending on system parameters, this results in the approximate range of (430–2860) for the total number of MD particles. The D3Q19 lattice Boltzmann implementation employs the parameters of grid size 1.0, a viscosity 1.0, thermal energy 1.0, particle density 1.0, time step 0.01 and coupling friction coefficient of 20.0. Unless specified, the results are all expressed in reduced MD units.

## 4. Conclusions

Reaction ensemble Monte Carlo simulations were used to study the ionization profile of a weak nanogel. Static nanogel properties were computed from these simulation data.

We find that the average dissociation state of the monomers in the nanogel increases towards the outside of the gel with increasing pH-p*K*_a_ value. However, this increase is modulated near the crosslinks where the average dissociation state is considerably lowered. These results are consistent with the idea that monomeric charges on a weak polyelectrolyte tend to be found on monomers that are statistically more distant from all other monomers. On the nanogel scale, this is expressed as charges accumulating near the nanogel surface. On the polymeric scale, this is expressed by having less charge near the crosslinks.

We find that salt concentrations affect the nanogel size in different ways depending on the value of pH-p*K*_a_. This is due to two competing effects arising from screening: increasing the screening will increase the mean nanogel charge, but will also decrease the electrostatic repulsion between charges.

Interestingly, when the nanogel size is plotted as a function of pH-p*K*_a_, all of the curves for the simulated salt concentrations cross at the same critical value of pH-p*K*_a_. More work is needed in order to ascertain whether this is a coincidence or if there exists a physical property to the nanogel that leads to the observed critical pH-p*K*_a_. When the nanogel size is plotted as a function of the dissociation fraction (or equivalently, the nanogel charge) the curves corresponding to different salt concentrations no longer cross, suggesting that the mean monomer charge is the relevant physical parameter in determining the size.

The total charge enclosed inside the nanogel is assessed by considering both monomeric and ionic contributions. We find that unsurprisingly, the internal charge increases with the monomeric charge and that increasing the salt concentration contributes to the reduction of the internal charge.

With the chemical equilibrium information computed using RxMC simulations (mainly the mean monomer charge), we use the pure MD with hydrodynamic interactions to investigate dynamical properties like diffusion and mobility.

The diffusion coefficient is expected to be related to the nanogel size: bigger nanogels have a lower diffusion coefficient. We find that the diffusion decreases with the mean charge fraction, which is consistent with the associated swelling observed from the reaction ensemble simulations.

The measured values for the electrophoretic mobility are leveling off surprisingly fast for increasing dissociation state. Beyond a dissociation state 〈α〉≈0.3, the mobility is almost constant with 〈α〉.

The addition of salt ions decreases the Debye length $\lambda_{D}$, which means that the local shear around the monomers occurs on a smaller length scale. The mobility thus decreases as the salt concentration is increased. Adding only 20 mMol of salt reduces the mobility of the simulated nanogel by roughly 35% compared to the salt-free case.

Our simulations provide qualitative insights into the dynamics of weak polyelectrolytes gels. We outlined possible challenges in treating the behavior of weak polyelectrolytes under the influence of an external electric field. Our results can serve as a stepping stone for upcoming models of weak polyelectrolyte dynamics.
